# Implants for trochanteric fractures in Norway: the role of the trochanteric stabilizing plate—a study on 20,902 fractures from the Norwegian hip fracture register 2011–2017

**DOI:** 10.1186/s13018-020-02163-x

**Published:** 2021-01-07

**Authors:** Carl Erik Alm, Frede Frihagen, Eva Dybvik, Kjell Matre, Jan Erik Madsen, Jan-Erik Gjertsen

**Affiliations:** 1 Division of Orthopedic Surgery, Oslo University Hospital, Oslo, Norway; 2 Institute of Clinical Medicine, Faculty of Medicine, University of Oslo, Oslo, Norway; 3 The Norwegian Hip Fracture Register, Department of Orthopedic Surgery, Haukeland University Hospital, Bergen, Norway; 4 Department of Clinical Medicine (K1), University of Bergen, Bergen, Norway

**Keywords:** Hip fractures, Trochanteric fractures, Trochanteric stabilizing plate, Intramedullary nail, Sliding hip screw, Internal fixation, Decision-making

## Abstract

**Background:**

The trochanteric stabilizing plate (TSP) is used as an adjunct to the sliding hip screw (SHS) in unstable trochanteric and subtrochanteric fractures. We wanted to describe the choice of implant for trochanteric fractures with a focus on the TSP in Norway.

**Methods:**

A total of 20,902 fractures from the Norwegian Hip Fracture Register treated surgically in 43 hospitals from 2011 to 2017 were included. Logistic regression analyses were performed to detect factors potentially influencing implant choice.

**Results:**

The mean age was 83 years, and 15,137 (72%) were women. An SHS was used in 13,273 (63%) fractures, of them 4407 (33%) with a TSP. Fracture classification was the most important determinant of TSP. In cases where an SHS was used, the odds ratio (OR) for using a TSP was 14 for AO/OTA 31A2 fractures and 71 for AO/OTA 31A3 and subtrochanteric fractures, compared to AO/OTA 31A1 fractures. The probability of receiving a TSP was higher in urban, academic, and high-volume hospitals (OR 1.2 to 1.3) and lower in Central and Northern Norway (OR 0.3 to 0.7). The use of an intramedullary nail (IMN) (*n* = 7629 (36%)) was also to a degree decided by fracture classification (OR 1.8 to 5.3). However, hospital factors, with OR 0.1 to 0.4 for IMN in academic, urban, and high-volume hospitals and OR 1.5 to 2.6 outside South-Eastern Norway (all *p* < 0.001), were also important.

**Conclusions:**

Fracture classification was the main determinant for TSP use. Any additional benefit from a TSP on postoperative fracture stability or clinical outcome needs to be clarified.

## Introduction

**Fig. 1:**
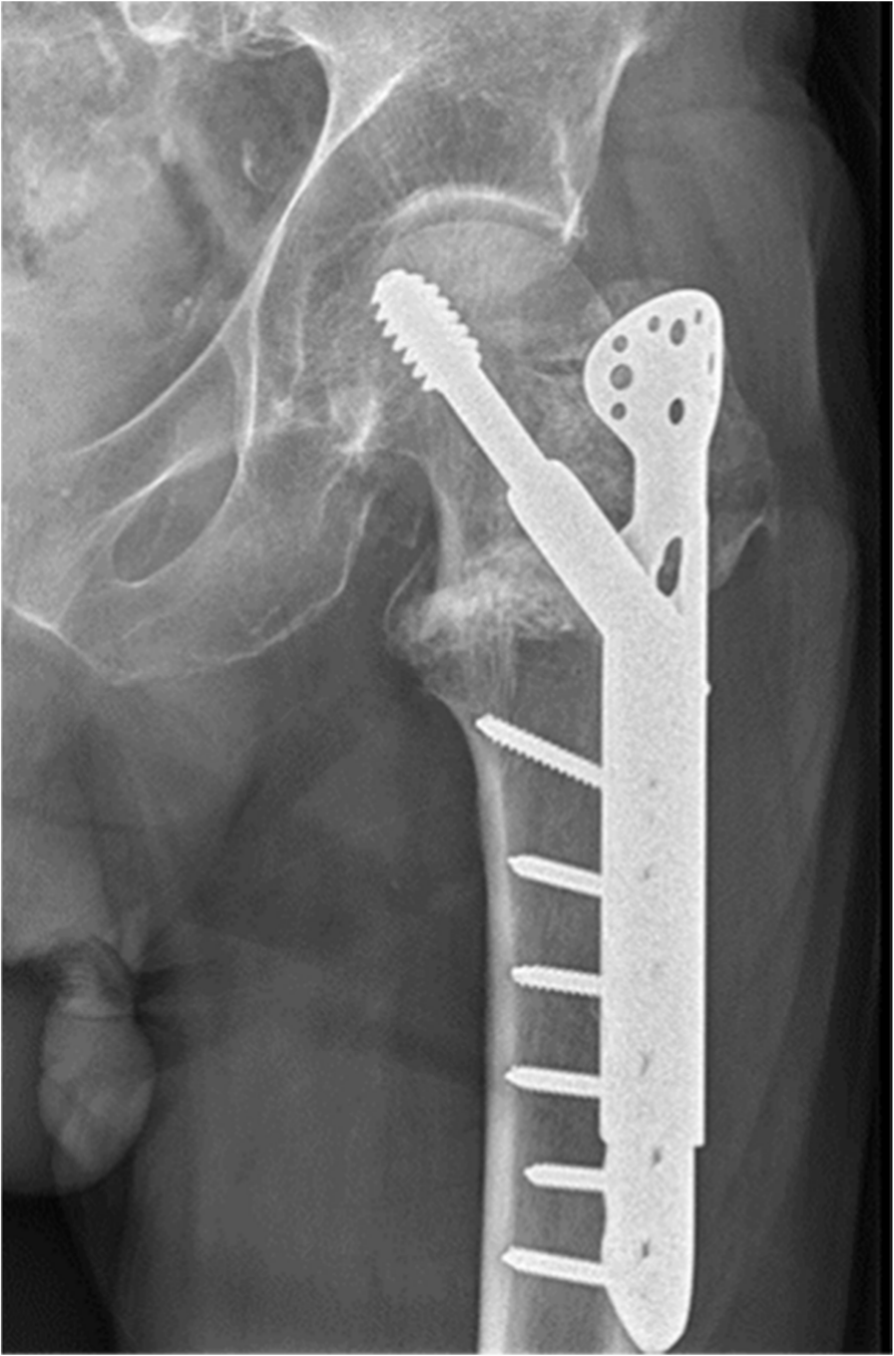
Radiograph of sliding hip screw with a modular trochanteric stabilizing plate in an AO/OTA 31A2 fracture (published with patient consent)

There is still an ongoing debate concerning the choice of implant for trochanteric fractures [1–3]. The literature on implant use is extensive, and studies comparing sliding hip screws (SHS) and intramedullary nails (IMN) are numerous [3–5]. The trochanteric stabilizing plate (TSP) is an extension of the SHS, most often modular (Fig. 1).

It functions as a lateral buttress and is added to increase stability and prevent medialization of the femoral shaft. Due to its modularity, it offers the possibility to enhance construct stability intraoperatively if required. The use of the TSP as an adjunct to the SHS varies between regions and hospitals, and the literature on the use and performance is scarce. Surgeon- and hospital-related factors may influence implant use, both in the absence of available evidence, and sometimes contrary to the evidence. The purpose of this study was to describe the use of the TSP in trochanteric and subtrochanteric fractures in Norway from 2011 to 2017. The secondary aim was to describe the use of IMN in the same period.

## Materials and methods

### Data

**Fig. 2:**
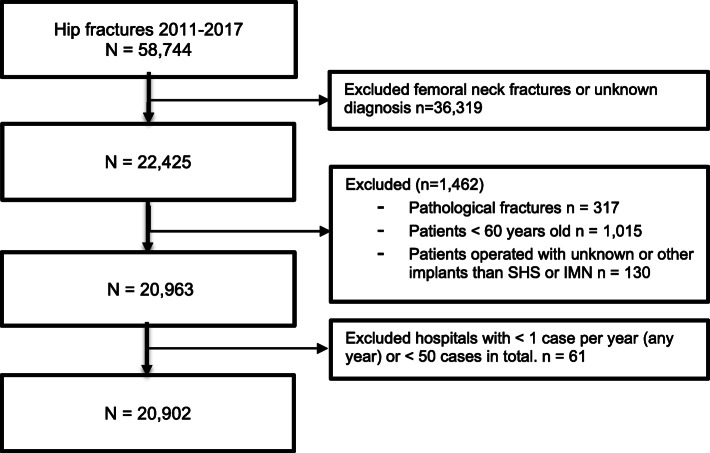
Flow chart of fractures included in the study

We applied prospectively registered data from the Norwegian Hip Fracture Register (NHFR) from 2011 to 2017. After the completion of the surgery, the surgeon reports information regarding the patient, the fracture, and the operation to the NHFR on a 1-page questionnaire. The completeness of reporting of primary procedures to the NHFR is 88% [6]. The dataset used in the primary analysis consisted of all trochanteric and subtrochanteric fractures registered from 2011 to 2017. Pathological fractures, fractures in patients < 60 years old, and fractures operated with other implants than SHS, SHS with TSP, or IMN were excluded from the analyses. In addition, hospitals were excluded if they either reported less than 50 cases to the NHFR in total during the study period or had at least 1 year where no fractures were reported (Fig. 2).

### Classification of hospitals

Hospitals were characterized as (1) situated in one of four national health regions; (2) low- or high-volume: less than 100 reported cases per year was considered low volume; (3) rural or urban: hospitals were classified as urban if they were situated in an urban area with more than 25,000 inhabitants; (4) academic or non-academic: academic hospitals had university affiliation and academic staff in the orthopedic department; and (5) in addition, hospitals were classified according to their surgical practice, i.e., implant choice. Hospitals where trochanteric fractures throughout the study period were treated with > 90% IMN or > 90% SHS were classified as “IMN hospitals” and “SHS hospitals,” respectively. Hospitals with a development from > 90% SHS to less than 80% during the period were classified as “SHS to mixed pattern hospitals.” Hospitals with a shift to > 90% IMN during the period were labeled “change to IMN hospitals.” Hospitals with none of these patterns were classified as “mixed pattern hospitals.”

### Statistics

The analysis of change of implant use over time was performed with chi-square comparing the different years. To analyze implant use, we performed two multivariate logistic regression analyses with either SHS with or without TSP, or SHS or IMN as dependent variables. Only fractures with complete datasets were included in the regression analyses. The variables for the regression analyses were selected a priori based on a presumed effect on implant choice, including patient, surgeon, and hospital factors. Data from the regression analyses are presented with odds ratio (OR), 95% confidence interval (CI) for OR, and *p* values. We used SPSS for Windows version 24 (IBM Corp, Armonk, NY, USA).

## Results

As of January 1, 2019, 20,902 fractures fulfilled the inclusion criteria and were included in the analyses (Fig. 2).

The mean age of patients was 83.2 years, 72% were women, and 26% had cognitive impairment (Table 1).

**Table 1:** Baseline characteristics, AO/OTA fracture classification, and surgeon experience by choice of implant

Implant	SHS	SHS with TSP	IMN	All fractures
Number, *n* (% of total)	8866 (42)	4407 (21)	7629 (36)	20,902
**Patient characteristics**				
Mean age (SD)	83.3 (8.6)	83.4 (8.8)	83.1 (8.7)	83.2 (8.7)
Women, *n* (%)	6241 (70)	3326 (75)	5570 (73)	15,137 (72)
ASA classes 1–2, *n* (%)	2972 (34)	1402 (32)	2543 (34)	6917 (34)
Cognitive impairment, *n* (%)	2395 (27)	1116 (25)	1879 (25)	5390 (26)
**Fracture type and surgeon experience**				
AO/OTA 31A1, *n* (%)	5807 (69)	450 (5)	2197 (26)	8454 (100)
AO/OTA 31A2, *n* (%)	2706 (31)	2901 (34)	2991 (35)	8598 (100)
AO/OTA 31A3 and subtrochanteric fractures, *n* (%)	353 (9)	1056 (27)	2441 (63)	3850 (100)
Surgeon experience > 3 years, *n* (%)	5520 (68)	2853 (71)	5860 (81)	14,233 (73)

**Fig. 3:**
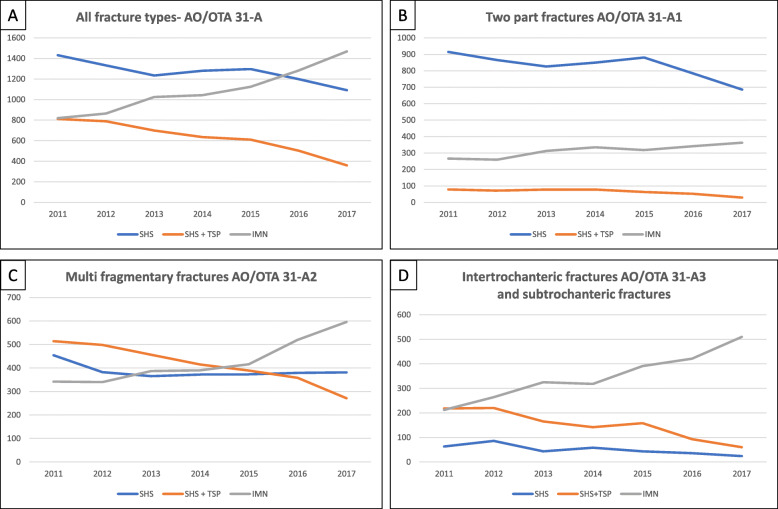
Time trend for the choice of implant. *Y*-axis—number of fractures. *X*-axis—year of operation. **a** All trochanteric and subtrochanteric fractures (*n* = 20,902). **b** Two-part trochanteric fractures (AO/OTA 31A1; *n* = 8454). **c** Multi-fragmentary trochanteric fractures (AO/OTA 31A2; *n* = 8598). **d** Inter- (AO/OTA 31A3) and subtrochanteric fractures (*n* = 3850)

The number of trochanteric fractures and the distribution of subgroups remained relatively stable throughout the period, ranging from 3090 fractures in 2017 to 3245 fractures in 2011 (Fig. 3).

The proportion of AO/OTA type A1 was 8454 fractures (40%), and of A2, there were 8598 fractures (41%). The number of A3 and subtrochanteric fractures as one group was 3850 (18%). The range of variation in fracture classification from 1 year to another was 37–43% A1, 39–42% A2, and 16–21% A3 and subtrochanteric fractures. The use of TSP declined throughout the period for all fracture types (*p* < 0.001) (Fig. 3). The use of IMN increased, especially in the unstable fracture patterns (*p* < 0.001). However, the SHS with or without the TSP remained the most used implant for simple two-part fractures (A1) (Fig. 3).

The classification of the 43 hospitals is shown in Table 2.

**Table 2:** Description of hospital-level implant choice and fracture proportion for trochanteric and subtrochanteric fractures

Hospital classification	Hospitals (***n***)	Fractures (***n***)	% of the total number of fractures
All hospitals	43	20,902	100
**Implant pattern**			
SHS hospitals	8	3808	18
IMN hospitals	6	3111	15
Change to IMN hospitals	7	2220	11
SHS to mixed pattern hospitals	13	8675	42
Mixed pattern hospitals	9	3088	15
**Hospital volume**			
High-volume (≥ 100 cases/year)	7	8086	39
Low-volume (< 100 cases/year)	36	12,816	61
**Location**			
Urban	18	13,750	66
Rural	25	7152	34
**Administrative “health region”**			
Southern and Eastern Norway	19	12,197	58
Western Norway	7	4080	20
Central Norway	8	2870	14
Northern Norway	9	1755	8
**Academic hospital**			
Academic	6	5218	25
Non-academic	37	15,684	75

Fourteen hospitals, reporting 1/3 of the fractures, were classified exclusively, or almost exclusively, as either SHS (8 hospitals) or IMN (6 hospitals) throughout the period. Twenty hospitals had a recognizable change in treatment practice during the period with an increased use of IMN. The largest group (13 hospitals, 42% of the fractures) changed from an exclusive use of SHS to a mixed pattern between SHS and IMN during the period. Seven hospitals (11% of the fractures), all “rural,” “low-volume,” and “non-academic” hospitals, were classified as “change to IMN hospitals,” i.e., an increase to > 90% intramedullary nailing of all fracture types during the study period. Three out of 6 “academic hospitals” (2702 fractures (13%)) were “SHS hospitals” throughout the period. The other three were classified as “SHS to mixed pattern hospitals.”

### Use of trochanteric stabilizing plate

A total of 13,272 fractures were operated with an SHS in the period, and an additional TSP was used in 4407 cases (33%). In stable type A1 fractures, the TSP was used in 7% of the SHS surgeries (450 out of 6257). For A2 fractures, the TSP was used in 52% of the SHS cases (2901 out of 5607), and in unstable A3 and subtrochanteric fractures, 75% of the patients had an additional TSP (1056 out of 1409). A total of 10,600 of these fractures could be included in the multivariate logistic regression analysis (Table 3).

**Table 3:** Logistic regression analysis of the use of trochanteric support plate (TSP) in adjunction to a sliding hip screw (SHS) depending on patient characteristics, fracture classification, and hospital characteristics. The analysis was performed on 10,600 cases operated with SHS (7308 (69%) without TSP and 3292 (31%) with TSP) with complete data

	OR for TSP	95% CI for OR	***p***
**Patient characteristics**			
Higher age (years)	1.0	0.99–1.0	0.9
Female sex	1.2	1.1–1.3	0.004
ASA > 2	1.1	1.0–1.2	0.05
Cognitive impairment (yes)	1.1	0.97–1.2	0.2
**Fracture type**			
AO/OTA 31A1	1		
AO/OTA 31A2	14	12–16	< 0.001
AO/OTA 31A3 and subtrochanteric	71	54–93	< 0.001
**Surgeon experience > 3 years**	0.9	0.8–1.0	0.05
**Hospital characteristics**			
Academic hospital	1.2	1.1–1.4	0.009
High-volume hospital (> 100 cases/year)	1.3	1.1–1.5	< 0.001
Urban hospital	1.2	1.0–1.4	0.01
Health region South-Eastern Norway	1		
Health region Western Norway	0.9	0.82–1.1	0.4
Health region Central Norway	0.3	0.25–0.35	< 0.001
Health region Northern Norway	0.7	0.52–0.83	< 0.001

Of these, 3292 fractures (31%) were operated with a TSP. Fracture classification (stability) was the most important determinant of whether a TSP was added or not, with OR 14 for type A2 fractures and OR 71 for A3 and subtrochanteric fractures. We found a statistically significant higher chance of getting a TSP in “academic,” “urban,” and “high-volume” hospitals. The probability of receiving a TSP was statistically significant less in Central and Northern Norway. Women had a small but statistically significant higher chance of receiving a TSP compared to men. Other patient factors and surgeon experience had limited impact. Overall, the 8 “SHS hospitals” operated 1308 of 3808 fractures (34%) with an SHS including a TSP, but the rate varied from 29 to 52% between hospitals (*p* < 0.001).

### Use of intramedullary nail versus sliding hip screw

We performed a separate regression analysis to evaluate determinants for the choice of SHS with or without a TSP compared to an IMN (Table 4).

**Table 4:** Logistic regression analysis of the use of intramedullary nail (IMN) versus sliding hip screw (SHS) with or without trochanteric support plate (TSP) on patient characteristics, fracture classification, and hospital characteristics. The analysis was performed on 15,655 (10,600 SHS and 5055 IMN) cases with complete data

Logistic regression SHS versus IMN	OR for IMN	95% CI for OR	***p***
**Patient characteristics**			
Higher age (years)	1.0	0.99–1.0	0.3
Female sex	1.0	0.89–1.1	0.6
ASA > 2	1.0	0.96–1.1	0.3
Cognitive impairment (yes)	1.1	0.98–1.2	0.1
**Fracture type**			
AO/OTA 31A1	1		
AO/OTA 31A2	1.8	1.6–1.9	< 0.001
AO/OTA 31A3 and subtrochanteric	5.3	4.5–6.2	< 0.001
**Surgeon experience > 3 years**	2.0	1.8–2.2	< 0.001
**Hospital characteristics**			
Academic hospital	0.1	0.08–0.11	< 0.001
High-volume hospital (> 100 cases/year)	0.4	0.35–0.44	< 0.001
Urban hospital	0.2	0.16–0.20	< 0.001
Health region South-Eastern Norway	1		
Health region Western Norway	2.6	2.3–3.0	< 0.001
Health region Central Norway	1.6	1.4–1.9	< 0.001
Health region Northern Norway	1.5	1.25–1.71	< 0.001

Fracture pattern was an important determinant for the use of IMN (OR 2 to 5). All variables describing hospital characteristics were statistically significant with low OR (0.1 to 0.4) for “academic,” “urban,” and “high-volume” hospitals. Analyses of the subgroups of hospitals according to hospital implant choice confirmed the pattern on hospital-related factors and fracture classification. For “mixed pattern” hospitals, the fracture classification was the dominant factor, and for “SHS hospitals” and “IMN hospitals,” the hospital-level factors were the most important (data not shown). Experienced surgeons, defined as surgeons having more than 3 years of experience with surgical fracture treatment, used IMN more frequently than less experienced surgeons (OR 2). No patient characteristics influenced the choice of implant significantly.

## Discussion

The use of the trochanteric stabilizing plate was mainly based on fracture classification and stability. Overall, a TSP was used in one of five fractures and in one of three cases where an SHS was used. The use of TSP declined during the study period, mainly due to the increased use of IMN. This change was most pronounced for unstable fractures. The use of IMN also depended on fracture stability, but hospital factors seemed to play a larger role. And overall, SHS was still the most frequently used implant, but its use towards the end of the study period was declining. An important reason for this change over time was that 13 hospitals abandoned the exclusive use of SHS in favor of IMN for unstable fracture patterns. In addition, 7 hospitals started to use IMN, for all or almost all fractures, regardless of fracture classification (Table 2). In addition to fracture stability, the south-east region and an academic and urban setting were associated with more frequent use of a TSP. Other than fracture classification, we did not identify patient factors relevant to the choice of implant. There was a strong influence of hospital policy on implant choice. This was supported by our ability to classify hospitals according to implant use. Twenty-eight hospitals representing 85% of the fractures used one implant for > 90% of the procedures during the whole period or part of the period. In contrast to the hospitals using one implant for all or almost all fractures, 9 hospitals had a mixed pattern between SHS and IMN throughout the period. In addition, 13 hospitals, representing 42% of the fractures, changed to a mixed implant pattern during the period. The mixed pattern may be a more evidence-based practice.

Most randomized trials comparing IMN and SHS or other implants for trochanteric fractures have been performed without the use of TSP or with the TSP used at the surgeon’s discretion in the SHS arm of the trial [3–5, 7]. The literature on TSP itself is scarce and mainly presenting low-level evidence. Four biomechanical studies examining the TSP in unstable trochanteric fracture models compared an SHS with TSP to an IMN, reporting similar mechanical properties between the two implants [8–11]. One paper compared SHS with and without TSP and reported less displacement on loading with the TSP [12]. Some clinical non-randomized comparisons exist, among them is a retrospective study showing less lag screw sliding and fewer reoperations in AO/OTA 31A2 fractures with the use of a TSP when the lateral wall thickness was below 2.24 cm [13]. Another study reported less lag screw sliding with TSP compared to SHS alone [14]. A register study of more than 3000 unstable fractures, although only 158 operated with TSP, found a tendency towards higher reoperation rates with SHS alone compared to SHS with TSP and IMN [15]. The only randomized trial examining the TSP directly compared SHS with and without TSP in 100 unstable fractures. No clinically relevant differences between the groups were found, neither in complications, secondary fracture displacement, nor functional results. This study, including 100 patients in total, may have been underpowered [16].

During our study period, we had no formal Norwegian guidelines for implant choice for trochanteric fractures. The Norwegian guidelines [17] (2018) recommends an SHS for AO/OTA type A1 fractures and IMN for subtrochanteric fractures. For A2 fractures, the main recommendation is an SHS with or without a TSP, with IMN as an option. For A3 fractures, IMN is recommended, but SHS with a TSP is stated as an option, depending on surgeon preference. An extrapolation of the Norwegian guidelines to the distribution of fractures reported in the present study should most likely lead to a “mixed practice” pattern. We have identified two other national guidelines discussing TSP for selected trochanteric fractures. Both German-Austrian guidelines [18] and Danish guidelines [19] discussed SHS with TSP as a possible alternative to IMN in unstable fractures. A more recent Danish algorithm did not include TSP as an option [20]. Other influential international guidelines, including from the American Academy of Orthopaedic Surgeons [21] and National Institute for Health and Care Excellence [22], do not mention the TSP. A review from the USA from 2004 recommended SHS for A1 and A2 fractures and SHS with TSP, or IMN for A3 fractures [23]. A more recent review from the same institution recommended SHS for A1 and possibly A2.1 fractures and IMN for other trochanteric and subtrochanteric fractures. The TSP was discussed as an option for unstable fractures in the latest review, but as an inferior implant compared to IMN [2].

When the evidence is unclear or may support various strategies, the choice of fracture treatment may depend on surgeon preferences, as well as local or national traditions [1, 24, 25]. In Norway, interestingly, we identified that implant choice is decided to a large degree on the hospital level and to some degree by the hospital circumstances (e.g., rural versus urban and regional differences). An increased use of IMN has been described in the USA, and both surgeon factors and hospital factors have been used to explain the choice of IMN instead of SHS [26]. A study based on Medicare data showed that younger surgeons used more nails. Nails were also more frequently used in high-volume teaching hospitals [24]. This contrasts with a survey among orthopedic surgeons in the USA where a non-academic setting was associated with IMN use, similar to our data [1]. The increased use of IMN in trochanteric fractures has also been reported in several studies from Europe [15, 27]. The increase of IMN in Norway with a parallel decrease in TSP use is thus in line with the development seen internationally.

Limitations of the present study include limited information on surgeons’ qualifications, and there may be surgeon-related variables influencing implant choice we have missed. A potential weakness is the lack of validation of the fracture classification in the register, but previous literature has indicated that the simple classification used here is reliable and independent of surgeon experience [28, 29]. A strength of this study is the large number of fractures with relevant clinical and administrative data. The NHFR has been validated, and a high completeness of reporting was found [6].

## Conclusion

In Norway, the TSP was used for unstable fracture types. In addition to fracture pattern, administrative and structural factors also explained the use of TSP, while patient factors played a limited role. The variation in the proportion of TSP between hospitals was considerable. We found a decreasing trend of TSP use, whereas IMN use increased. The ability of the TSP to avoid fixation failure or provide better clinical outcomes remains unclear, and larger studies addressing these issues are warranted.

## Data Availability

The data that support the findings of this study are available from The Norwegian Hip Fracture Register, but restrictions apply to the availability of these data, which were used under license for the current study, and so are not publicly available. Data are however available from the authors upon reasonable request and with permission from The Norwegian Hip Fracture Register.

## Abbreviations

TSP: Trochanteric stabilizing plate
SHS: Sliding hip screw
IMN: Intramedullary nail
NHFR: Norwegian Hip Fracture Register
OR: Odds ratio
SD: Standard deviation
CI: Confidence interval
